# Micro&Nano2010 - Special Symposium on Nanomaterials for sensing and energy harvesting devices

**DOI:** 10.1186/1556-276X-6-244

**Published:** 2011-03-22

**Authors:** Androula G Nassiopoulou

**Affiliations:** 1 NCSR Demokritos, Institute of Microelectronics (IMEL), Terma Patriarchou Grigoriou, Aghia Paraskevi, 153 10 Athens, GREECE

## 

This special issue of Nanoscale Research Letters contains scientific contributions presented at the International Conference "Micro&Nano2010 - Special Symposium on Nanomaterials for sensing and energy harvesting devices", which was held in Athens, Greece, from December 12-15, 2010.

Micro&Nano2010 was the fourth of a series of Conferences on Microelectronics, Nanoelectronics, Nanotechnologies and MEMs held in Athens-Greece every three years and organized by the "Micro&Nano" Scientific Society (http://www.micro-nano.gr). The Conference aims at gathering together in an interactive forum scientists and engineers working in the challenging field of Microelectronics, Nanoelectronics, Nanotechnologies and MEMs and to stimulate discussions in last achievements and new developments in this rapidly evolving field. One of the key objectives of the Conference is to promote collaboration and partnership between different academia, research and industry players in the field. The Conference combines an extensive scientific programme, including oral and poster sessions, with an exhibition and social events.

Within "Micro&Nano2010" a special Symposium was organized on nanomaterials for sensing and energy harvesting devices. A selected collection of papers of this Symposium are found in the present special issue. The full volume of the Proceedings, including micro- and nanomaterials, technologies and devices, will appear as a special issue of the journal "Microelectronic Engineering".

The guest editor of this issue and Chairperson of "Micro&Nano2010"

Androula G. Nassiopoulou

Head of the Nanostructures for Nanoelectronics, Photonics and Sensors Research group

Terma Patriarchou Grigoriou, Aghia Paraskevi

153 10 Athens, Greece

President of Micro&Nano Scientific Society

Director of Research, IMEL/NCSR Demokritos

e-mail: A.Nassiopoulou@imel.demokritos.gr

